# A Quantitative Dynamic Simulation of *Bremia lactucae* Airborne Conidia Concentration above a Lettuce Canopy

**DOI:** 10.1371/journal.pone.0144573

**Published:** 2016-03-08

**Authors:** Mamadou Lamine Fall, Hervé Van der Heyden, Odile Carisse

**Affiliations:** 1 Biology Department, University of Sherbrooke, 2500 De l’Université Blvd., Sherbrooke, QC, Canada, J1K 2R1; 2 Compagnie de Recherche Phytodata inc., 111 Rang Saint-Patrice, Sherrington, QC, Canada, J0L 2N0; 3 Horticulture Research and Development Centre, Agriculture and Agri-Food Canada, 430 Gouin Blvd., St-Jean-sur-Richelieu, QC, Canada, J3B 3E6; Leibniz-Institute of Vegetable and Ornamental Crops, GERMANY

## Abstract

Lettuce downy mildew, caused by the oomycete *Bremia lactucae* Regel, is a major threat to lettuce production worldwide. Lettuce downy mildew is a polycyclic disease driven by airborne spores. A weather-based dynamic simulation model for *B*. *lactucae* airborne spores was developed to simulate the aerobiological characteristics of the pathogen. The model was built using the STELLA platform by following the system dynamics methodology. The model was developed using published equations describing disease subprocesses (e.g., sporulation) and assembled knowledge of the interactions among pathogen, host, and weather. The model was evaluated with four years of independent data by comparing model simulations with observations of hourly and daily airborne spore concentrations. The results show an accurate simulation of the trend and shape of *B*. *lactucae* temporal dynamics of airborne spore concentration. The model simulated hourly and daily peaks in airborne spore concentrations. More than 95% of the simulation runs, the daily-simulated airborne conidia concentration was 0 when airborne conidia were not observed. Also, the relationship between the simulated and the observed airborne spores was linear. In more than 94% of the simulation runs, the proportion of the linear variation in the hourly-observed values explained by the variation in the hourly-simulated values was greater than 0.7 in all years except one. Most of the errors came from the deviation from the 1:1 line, and the proportion of errors due to the model bias was low. This model is the only dynamic model developed to mimic the dynamics of airborne inoculum and represents an initial step towards improved lettuce downy mildew understanding, forecasting and management.

## Introduction

Lettuce (*Lactuca sativa* L.) downy mildew, caused by the oomycete *Bremia lactucae* Regel, is a major threat to lettuce production around the world [1–2]. The life cycle of *B*. *lactucae*, an obligate biotrophic parasite, involves primary (sexual) and secondary (asexual) infection cycles. The sexual cycle results in soilborne oospores that are the likely source of primary inoculum [3]. Oospores can potentially overwinter and become a source of primary inoculum during the spring. However, there are no published data showing that *B*. *lactucae* oospores survive the harsh Canadian winter. The asexual cycle generates conidia that are adapted to aerial dispersal [4–5]. The conidia are produced when humidity is high and wind speed is low, and they are released in response to concomitant decreasing humidity and increasing temperature [6]. Viable conidia that land on leaves of susceptible lettuce plants germinate and colonize the leaves, resulting in symptoms that are visible 7 to 14 d after the initiation of infection [7]. First infection is then followed by successive asexual cycles occurring throughout the lettuce production season. Environmental factors such as temperature, relative humidity (RH), wind speed, solar radiation, and leaf wetness duration have been identified as factors that determine the extent of conidia production, dispersal, and survival as well as the infection processes [4, 6, 8, 9, 10]. Hence, conidia survival is greater at 23°C (12h) than at 31°C (2 to 5 h), regardless of RH (33% and 76%), and conidia survival increases substantially at an RH of 90% or greater [5, 9]. Wind speed plays a major role in the conidia dispersal process, and solar radiation determines the survival of the airborne conidia [5, 7]. However, the most important factor for successful infection is the duration of morning and evening leaf wetness [11–15].

The control strategy for lettuce downy mildew is based mostly on chemical protection with fungicide applied at fixed or weather-based intervals. A difficulty that most growers face is identifying the best timing for fungicide applications to get the optimum level of control against downy mildew. Therefore, in the province of Quebec, Canada, fungicides are applied routinely to control downy mildew even though some of these applications may be unnecessary. In the pathosystem formed by lettuce and *B*. *lactucae*, two decision-support systems (DSSs) were developed to guide decision-making concerning the timing of fungicide applications. The first DSS, which was developed in California, USA, and was later modified, is based on leaf wetness ending late in the morning (10:00 hours) to predict when infection by *B*. *lactucae* occurs [13]. The second DSS, BREMCAST, was developed in Quebec, Canada [12], use a leaf wetness duration of 3 to 5 h after dawn (continuing until 10:00 hours) as an action threshold for fungicide application. Therefore, in both of these DSSs, leaf wetness duration is used as an indicator for occurrence of an infection event [12–13, 16]. These systems assume that sporulation is nocturnal, that conidia are released at dawn, and that infections occur in the morning [12–13]. However, the major limitation of these DSSs is their inability to assess the presence and amount of conidia above lettuce fields, and consequently these systems can overestimate or underestimated the risk of downy mildew infection [17]. In fact, to evaluate the availability of *B*. *lactucae* airborne inoculum, these DSSs rely on signs of downy mildew in the field during routine scouting [12].

The potential risk of lettuce downy mildew development and consequent yield losses are related to the quantity of *B*. *lactucae* airborne conidia [15]. Fall et al. [15] observed a quantitative relationship between the airborne conidia concentration (ACC) of *B*. *lactucae* and the number of lesions per leaf. Hence, an ACC of 14 conidia/m^3^ can cause one lesion per leaf [15]. Therefore, incorporating *B*. *lactucae* airborne conidia concentration into DSSs may help to develop more effective strategies for controlling lettuce downy mildew with better timing of fungicide applications [15].

The amount of airborne conidia above lettuce fields is dictated by several factors, including the sporulation intensity of sporulating lesions (source), the proportion of conidia that is released, the proportion of conidia that escapes the canopy layer and becomes airborne, and the proportion of surviving conidia that are deposited on susceptible lettuce leaves. Because each of these factors has been mathematically described for *B*. *lactucae* or closely related species [12–13, 18–19], these mathematical relationships could be used to simulate the quantity of airborne conidia.

Despite the amount of scientific information available on the aerobiology of plant pathogens, few published studies have actually focused on the simulation of airborne inoculum, even though it has been documented that inoculum is a key factor for the development of epidemics of several plant diseases [20–22].

In this study, we developed a dynamic simulation model of *B*. *lactucae* airborne conidia in an effort to improve the decision-making process for lettuce downy mildew management. The specific objectives of this study were (1) to develop a structurally dynamic model to simulate *B*. *lactucae* airborne conidia and (2) to assess the accuracy and sensitivity of the model against measured airborne conidia data.

## Materials and methods

### Ethics statement

The study did not involve endangered or protected species. All data are available in Tables, Figures and Supporting Information files.

### Asexual disease cycle processes

**Fig 1:**
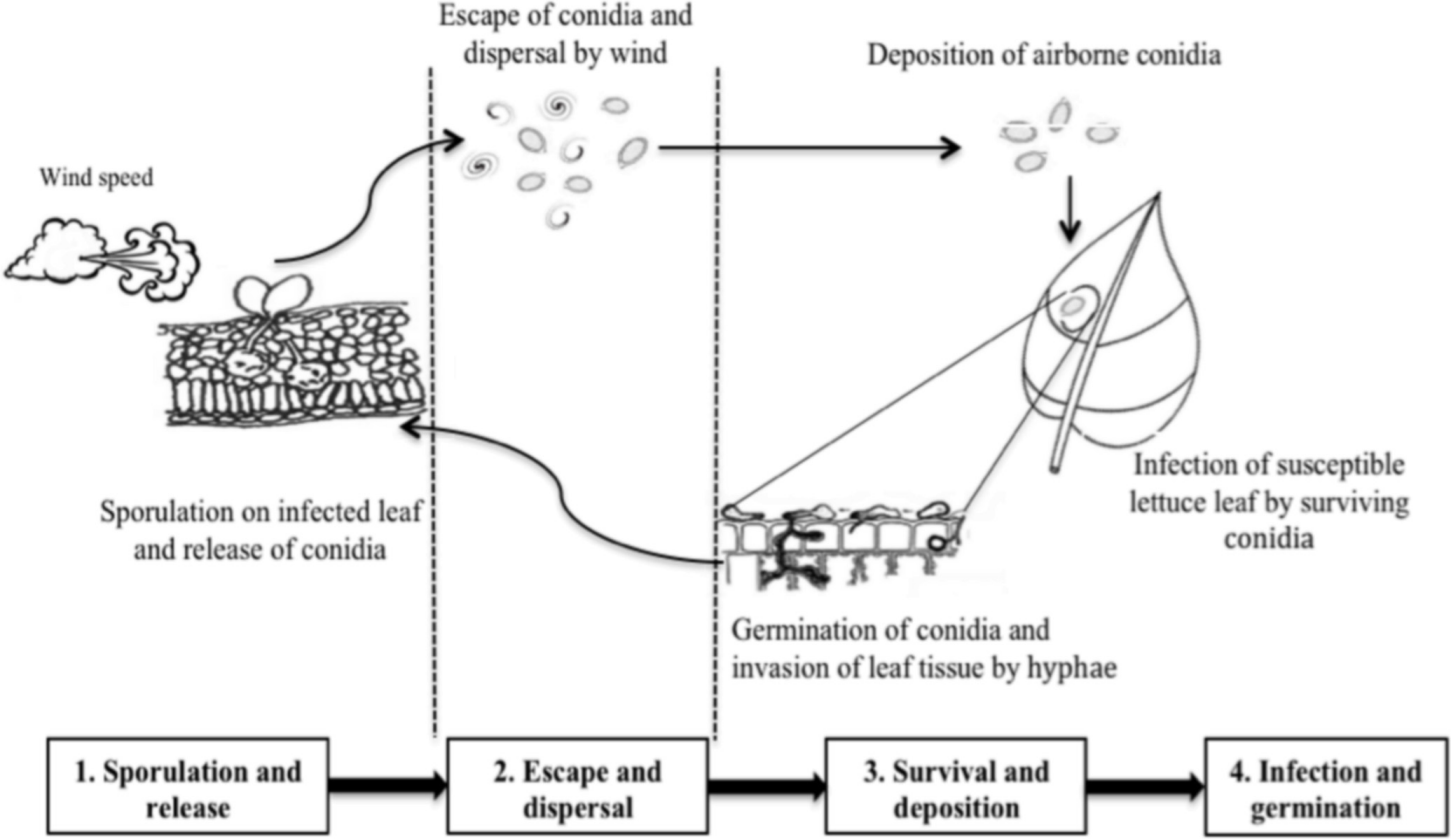
Asexual stages of the life cycle of *Bremia lactucae*.

Because of the absence of information and evidence about the overwintering of oospores under Canadian weather conditions, the asexual life cycle of *B*. *lactucae* was used to build the model framework. This cycle can be divided in four major stages: the sporulation and release stage, the escape and dispersal stage, the survival and deposition stage, and the infection and germination stage. First, conidia that land on the leaves of susceptible lettuce plants germinate and colonize the leaf cells. The resulting infected leaves produce conidia under conditions of high humidity and low wind speed. These conidia are released in response to decreasing humidity and increasing temperature (sporulation and release stage). Second, increases in wind speed promote the escape of conidia from the canopy layer and the dissemination of these conidia (escape and dispersal stage). Third, weather variables, including solar radiation, temperature, and air RH, determine conidia survival, and as the wind speed decreases, the conidia are deposited gradually on lettuce leaves (survival and deposition stage). Finally, new infections by conidia require free water on leaf surfaces for germination (infection and germination stage) [8, 15]. Fig 1 shows the different stages of the asexual life cycle of *B*. *lactucae*.

### Modeling approach

System dynamics is a methodology for studying and managing complex systems that change over time [23]. A model was built using the system dynamics methodology to simulate the asexual life cycle of *B*. *lactucae*. The model was developed according to the principles of “flow charts” in a dynamic simulation system (STELLA, v. 10.6.0).

STELLA is a flexible computer modeling package with an easy, intuitive interface that allows users to construct dynamic models that can simulate biological systems. The main components of STELLA are stocks, flows, connectors, and converters. Stocks are accumulations within the system and can be different types, including reservoirs and conveyors. Flows are the movement of the stocks throughout the system and allow resources to be transported around the model. Connectors provide information links within the system, and converters contain the algebraic relationships within a model [23]. The change in any stock at a given time is expressed as follows:

Stocks(t)=Stocks(t−dt)+(n*Inflow−n*outflow)dt (1)

where *t* is time, *dt* is differential of *t*, and *n* is number of inflows or outflows

The simulation time unit is hour, and the variable inputs are estimated on an hourly basis. To reduce potential integration error, a built-in simulation algorithm of the Euler integration method and a simulation time step of 0.25 are used [23]. As a result, for each hour of simulation, the STELLA program runs four times for the integration process. The time horizon of the model is 24 h (1 d), and the model simulates the airborne conidia of *B*. *lactucae* from July to September of each year, which is the critical period for lettuce downy mildew in Quebec, Canada.

### Model description

**Fig 2:**
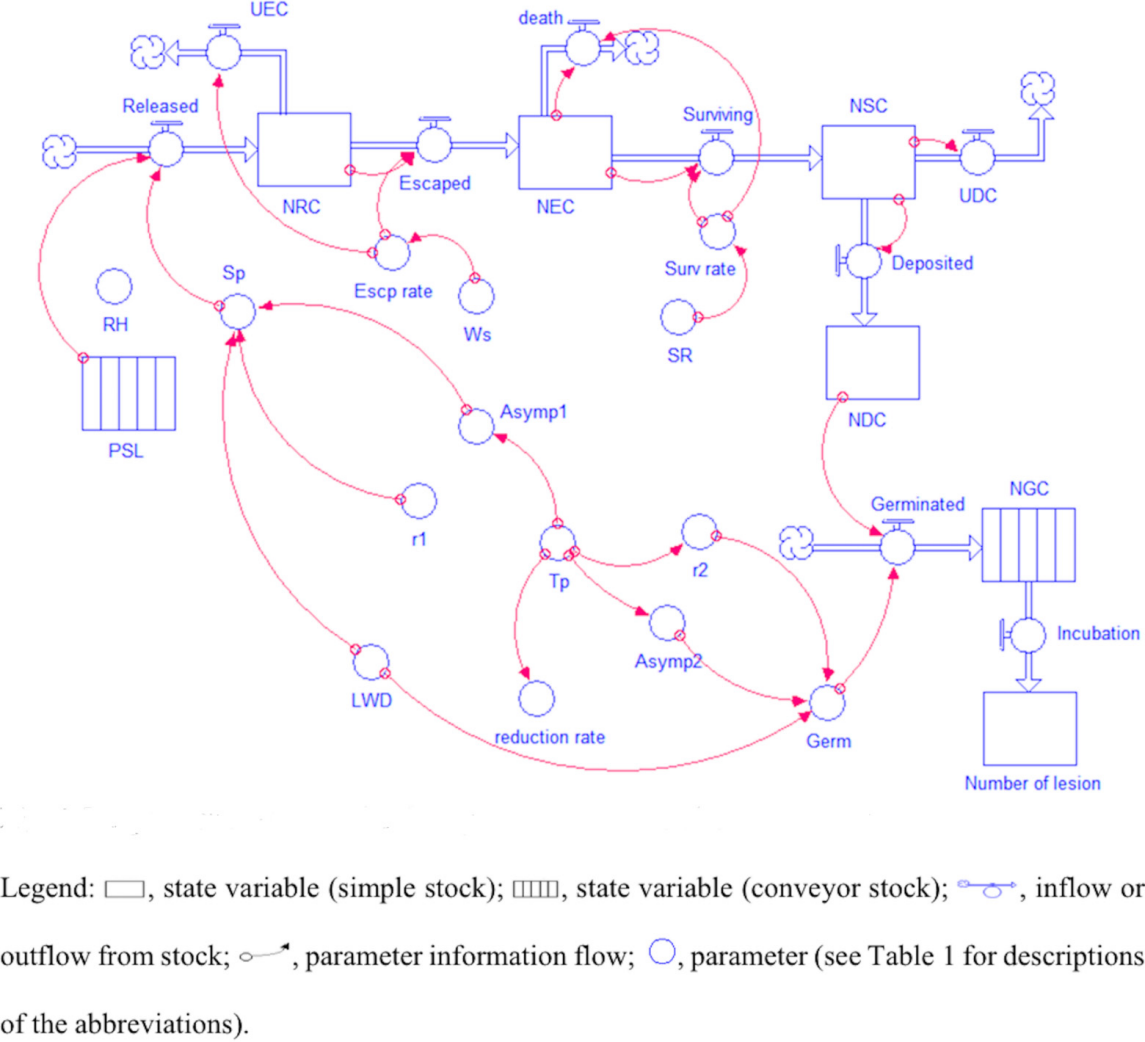
Diagram of the model simulating *Bremia lactucae* airborne conidia.

The model can be divided into six state variables (stocks) that are linked by flow charts and connectors (Fig 2, Table 1). The first state variable is the potential for the sporulation of one lesion (PSL). It is assumed that at least one lesion per m^2^ of crop is sporulating when the temperature is between 5 and 25°C and the leaf wetness duration is greater than or equal to 2 h [15, 24]. It is also assumed that, for the lesion lifetime and for the time frame of July to September, a sporulating lesion can produce a maximum of 20,000 conidia (unpublished data) and that the mean lesion size is 0.004 m^2^ [25].

**Table 1:** Description of variables and parameters used in the model.

Abbreviation	Description	Unit
**PSL**	Potential for the sporulation of one lesion per m^2^	Number (= 1)
**NRC**	Number of released conidia per m^2^	Number (0 to ∞)
**NEC**	Number of escaped conidia per m^2^	Number (0 to ∞)
**NSC**	Number of survived conidia per m^3^	Number (0 to ∞)
**NDC**	Number of deposited conidia per m^2^	Number (0 to ∞)
**NGC**	Number of germinated conidia per m^2^	Number (0 to ∞)
**Sp**	Sporulation	Number (0 to ∞)
**Escp rate**	Escape rate	Number (0 to ∞)
**Surv rate**	Survival rate	Number (0 to ∞)
**Germ**	Germination	Number (0 to ∞)
**UEC**	Unescaped conidia	Number (0 to ∞)
**UDC**	Undeposited conidia	Number (0 to ∞)
**Asymp1**	Asymptote for sporulation equation	Number (0 to ∞)
**Asymp2**	Asymptote for germination equation	Number (0 to ∞)
**r1**	Rate for sporulation equation	0.75
**r2**	Rate for germination equation	Number (0 to ∞)
**Ws**	Wind speed	m/s
**SR**	Solar radiation	MJ/m^2^
**RH**	Relative humidity	%
**LWD**	Leaf wetness duration	H
**Tp**	Average temperature over leaf wetness duration	°C

The second state variable is the number of released conidia (NRC). The change in NRC is dictated by the conidia inflow (released conidia), the conidia outflow (escaped conidia), and the conidia death rate, as follows:

NRC(t)=NRC(t−dt)+(Released−Escaped−Unescaped conidia)dt (2)

The algebraic equations describing conidia inflow and conidia outflow for Eq 2 are defined in Table 2.

**Table 2:** Algebraic equations describing the movement of the stocks throughout the model.

**Variable**	Equation	Data source
**Sp**	*Asymp*1(1 + 25118.86 × exp(−*r*1 × *LWD*))^−0.909^	Tchervenivanova, 1995 [18]
**Asymp1**	0.996 – 0.000051 × *Tp*^3^ – 15.575/(*Tp*)^2^	Tchervenivanova, 1995 [18]
**Germ**	*Asymp*2 × exp(−exp(*r*2 × (*LWD* − 2)))	Scherm and van Bruggen, 1993 [16]
**Asymp2**	0.385 + 0.054 × *Tp* − 0.0024 × *Tp*^2^	Scherm and van Bruggen, 1993[16]
**r2**	−1.154 + 0.327 × *Tp* − 0.011 × *Tp*^2^	Scherm and van Bruggen, 1993 [16]
**Escp rate**	0.073 × *Ws* − 0.0087	Fall, unpublished data, 2013
**Surv rate**	IF SR<1 THEN 0.8 ELSE 0.6	Bhaskara Reddy et al., 1996 [26]
**Released**	*PSL* × *Sp*	
**Escaped**	*NRC* × *Esca*. *rate*	
**Surviving**	*NEC* × *Surv*. *rate*	
**Deposited**	*NSC* × 1/3	Kranz, 1974 [27]
**Germinated**	*NDC* × *Germ*	
**Incubation**	240 h	Scherm and van Bruggen,1993, 1994 [8, 16]

The third state variable is the number of escaped conidia (NEC). The change in NEC is dictated by the conidia inflow (escaped conidia), the conidia outflow (surviving conidia), and the conidia death rate, as follows:

NEC(t)=NEC(t−dt)+(Escaped−Surviving−Conidia deaths)dt (3)

The algebraic equations describing conidia inflow and outflow for Eq 3 are defined in Table 2.

The fourth state variable is the number of surviving conidia (NSC). The change in NSC is a function of conidia survival and conidia deposition, as follows:

NSC(t)=NSC(t−dt)+(Surviving−Deposited−Undeposited conidia)dt (4)

The fifth state variable is the number of deposited conidia (NDC), described as follows:

NDC(t)=NDC(t−dt)+(Deposited)dt (5)

where the variables are defined in Table 2.

The sixth state variable is the number of germinated conidia (NGC), described as follows:

NGC(t)=NGC(t−dt)+(Germinated−Incubation)dt (6)

The germination process and the duration of incubation dictate the changes in the NGC. The duration of incubation is defined as the time from spore deposition to lesion production and is described in Table 2.

Running the model requires weather variables, including leaf wetness duration, RH, temperature, wind speed, and solar radiation (Table 1). Each of the six state variables can be chosen as model outputs.

### Measurement of environmental variables

Leaf wetness duration was assessed every 15 minutes with electrical-impedance leaf-wetness sensors (Model 237; Campbell Scientific, Edmonton, AB, Canada) placed at the height of the lettuce leaves. In 1997 and 1998, air temperature (°C) was recorded with a data logger (Model 21X; Campbell Scientific) placed near the spore sampler, and RH (%) was monitored with a probe (Model HMP35C; Campbell Scientific). In 2003 and 2004, air temperature and RH were monitored using WatchDog data loggers (Spectrum Technologies, Aurora, IL, USA) placed near the spore sampler. Weather variables were monitored every 30 min, and hourly averages were used in the analyses. The temperature and RH probes were placed in a white shelter 1.5 m above the ground. For all years, wind speed (10 m above the ground) and solar radiation data were obtained from an Environment Canada weather station located approximately 200 m from the plots [7, 15].

### Model evaluation

The model was evaluated using four years (1997, 1998, 2003, and 2004) of hourly data on *B*. *lactucae* ACC [7, 15]. The data were collected between 1 July and 20 September in each of 1998, 1997, 2003, and 2004. Each year, a plot of the lettuce cultivar Ithaca was established in an organic soil at the Agriculture and Agri-Food Canada experimental farm in Ste-Clotilde, QC, Canada (latitude 45°10′ N, longitude 73°40′ W). Lettuce plants produced in a greenhouse by Les Serres Lefort (Ste-Clotilde, QC, Canada) were transplanted 0.3 m apart in the rows, with 0.35 m between rows. A 7-d volumetric spore sampler (0.94 height, Standard orifice 0.002 m x 0.014 m, Burkard Manufacturing Co., Rickmansworth, Hertfordshire, UK) placed in the center of the plot was used to monitor ACC two weeks per month (alternating sampling weeks, one on, one off) between July and September. The sampler was adjusted to sample air at 0.01 m^3^/min. Impaction tapes were coated with a thin layer of silicone grease before they were placed in the sampler. Tapes were removed at 7-days intervals, cut into 0.048 m long segments corresponding to 24 h periods. Conidia were counted on whole transects perpendicular to the tape length. These transects were fixed at 0.002 m intervals to obtain hourly counts. Conidia counts were performed with a microscope at 250× magnification (0.00075 m wide transect) and converted to conidia per cubic meter of air for each hour of the day [7].

For each year, the hourly-simulated conidia concentrations were compared with the hourly-observed conidia concentrations for a period of 24 h. The cumulative conidia concentration within a 24-h period was considered the daily conidia concentration, and hence the daily-simulated conidia concentrations were compared with the daily-observed conidia concentrations. In order for accuracy to be compared, regression of observed data (on the y-axis) versus simulated data (on the x-axis) was used instead of regression of simulated data (on the y-axis) versus observed data (on the x-axis) [28–29]. Also, when the coefficient of determination was greater than 0.7, Theil’s decomposition of error was performed [30]. Theil’s U statistic is decomposed into three coefficients of inequality (Eq 7): mean differences between the observed and simulated airborne conidia, U_bias_; deviations from the 1:1 line, U_slope_; and the unexplained variance, U_error_. The coefficients are calculated as follows:

$$U_{bias}=\;\frac{n{\left(\bar{O}-\bar{S}\right)}^{2}}{\sum {\left(O-S\right)}^{2}};U_{slope}=\;\frac{{\left(b-1\right)}^{2}\sum {\left(S-\bar{S}\right)}^{2}}{\sum {\left(O-S\right)}^{2}};U_{error}=\;\frac{\sum {\left(\tilde{O}-O\right)}^{2}}{\sum {\left(O-S\right)}^{2}}$$ (7)

where *n* is the number of simulations, *O* and *S* are the observed and simulated values of airborne conidia, respectively, $\bar{O}$ and $\bar{S}$ are the observed and simulated means, respectively, and *b* is the slope in the equation $\tilde{O}=a+bS$, where $\tilde{O}$ is calculated observed values based on simulated values. The sum of the three coefficients is 1.

A numerical sensitivity analysis was carried to describe how much the model output values (simulated number of airborne conidia concentrations) are affected by the changes in the model input values (leaf wetness duration, sporulation rate [which is defined as a function of temperature; see Table 2], wind speed, solar radiation, and RH). To evaluate how increasing one unit at time, of each input, will affect the increase in the simulated conidia number, the inputs variables were gradually increased and the corresponding airborne conidia concentrations were measured in each simulation run.

## Results

**Fig 3:**
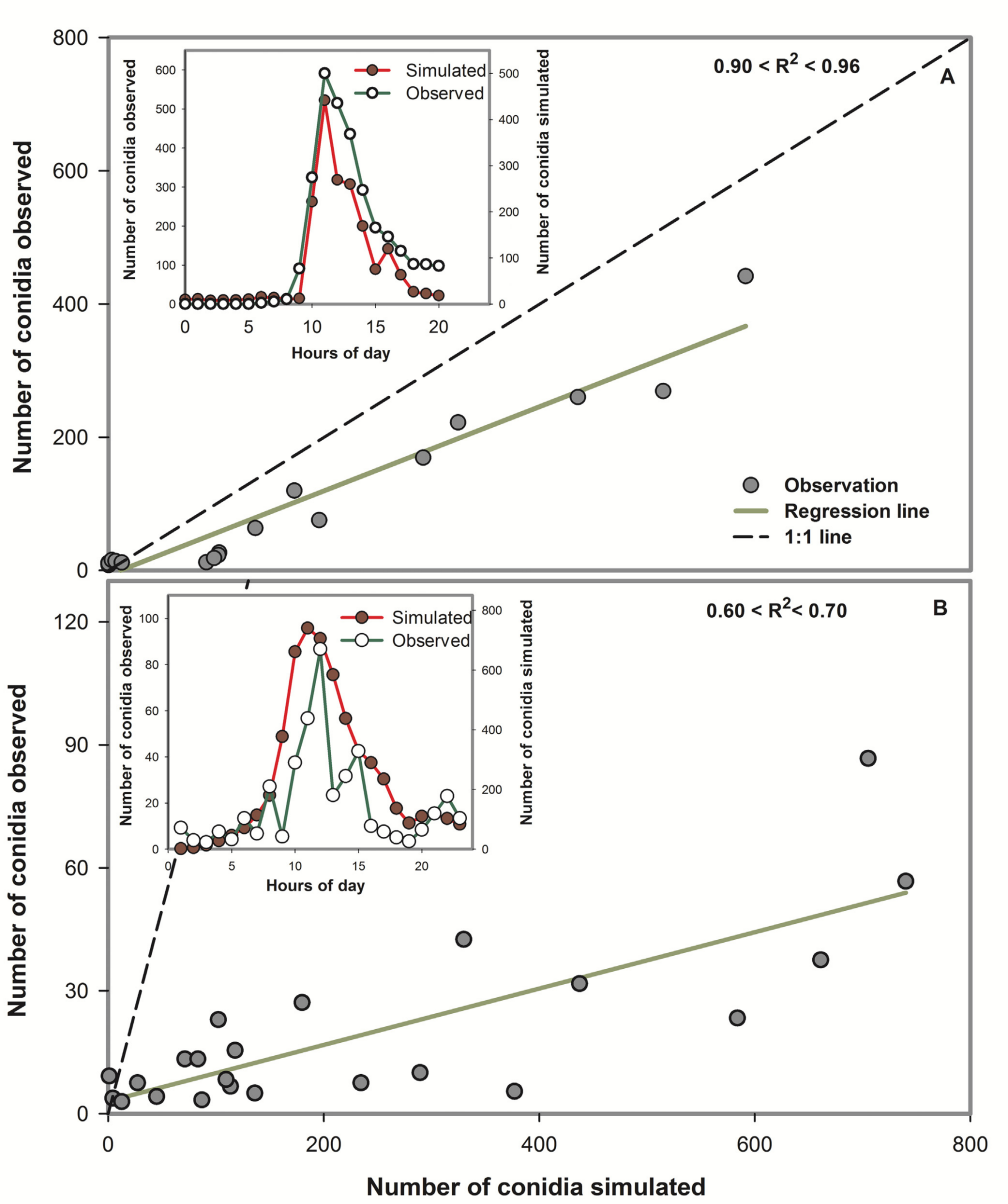
Examples of scatter plots of hourly-simulated versus hourly-observed airborne conidia concentrations of *Bremia lactucae* for the dynamic model developed to monitor airborne inoculum of the lettuce downy mildew pathogen. (A) Example of a case when 0.90 < *R*^2^ < 0.96, and (B) example of case when 0.60 < *R*^2^ < 0.70. The dashed line indicates 1:1 agreement between simulated and observed airborne conidia. The solid line indicates the fitted values from the regression of simulated versus observed airborne conidia. *R*^2^, coefficient of determination. The inset graph in each panel represents the hourly-simulated and hourly-observed airborne conidia as a function of hour of day.

**Fig 4:**
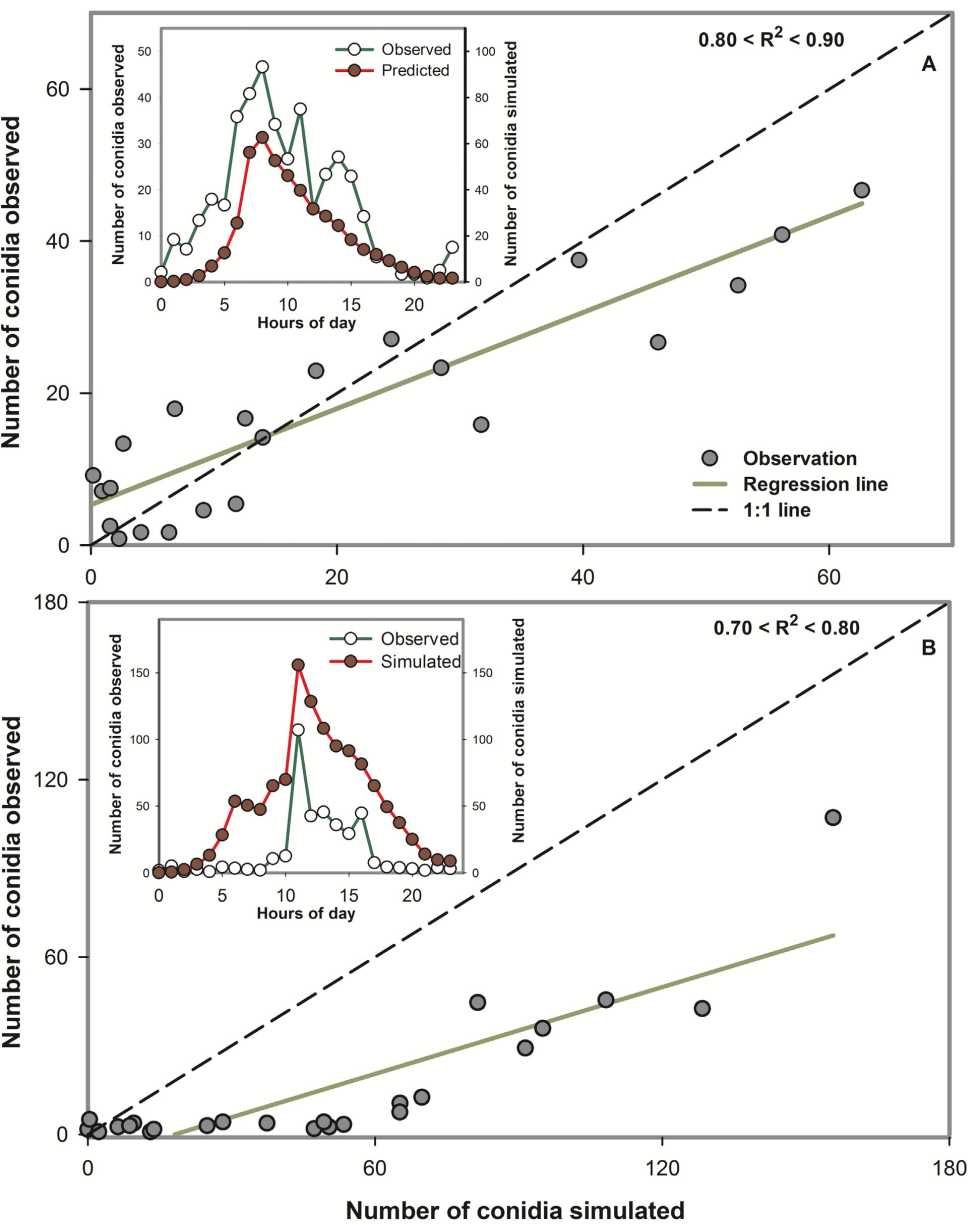
Examples of scatter plots of hourly-simulated versus hourly-observed airborne conidia concentrations of *Bremia lactucae* for the dynamic model developed to monitor airborne inoculum of the lettuce downy mildew pathogen. (A) Example of a case when 0.80 < *R*^2^ < 0.90, and (B) example of a case when 0.70 < *R*^2^ < 0.80. The dashed line indicates 1:1 agreement between simulated and observed airborne conidia. The solid line indicates the fitted values from the regression of simulated versus observed airborne conidia. *R*^2^, coefficient of determination. The inset graph in each panel represents the hourly-simulated and hourly-observed airborne conidia as a function of hour of day.

In the time window from July to September in 1997, 1998, 2003, and 2004, a total of 132 simulation runs were performed. Simulation runs (one run correspond to one day) were done 32, 36, 33, and 31 times in 1997, 1998, 2003, and 2004 respectively. There was a linear relationship between the hourly-observed number of conidia and the hourly-simulated number of conidia. In 1997, 1998, 2003, and 2004, the proportion of the linear variation in the hourly-observed values explained by the variation in the hourly-simulated values was greater than 0.7 (*R*^2^) in 32.5%, 94.7%, 96.8%, and 100% of the total number of simulated days, respectively (Table 3, Figs 3 and 4). The hourly-simulated and observed conidia concentrations had a similar trend and shape (Figs 3 and 4).

**Table 3:** Coefficients of determination of the regression model of hourly-observed versus hourly-simulated airborne conidia concentrations of *Bremia lactucae* in a lettuce field. n, number of simulations; R2, coefficient of determination.

Year	*n*	Coefficients of determination				
		*R*^2^ > 0.9	0.8 <*R*^2^ <0.9	0.7 < *R*^2^ < 0.8	0.6 < *R*^2^ < 0.7	*R*^2^ < 0.6
**1997**	32	10.4%	15.3%	6.8%	36.8%	30.6%
**1998**	36	7.5%	10.6%	76.6%	5.3%	0.0%
**2003**	33	15.2%	16.3%	65.3%	3.0%	0.0%
**2004**	31	5.0%	30.9%	64.1%	0.0%	0.0%

**Fig 5:**
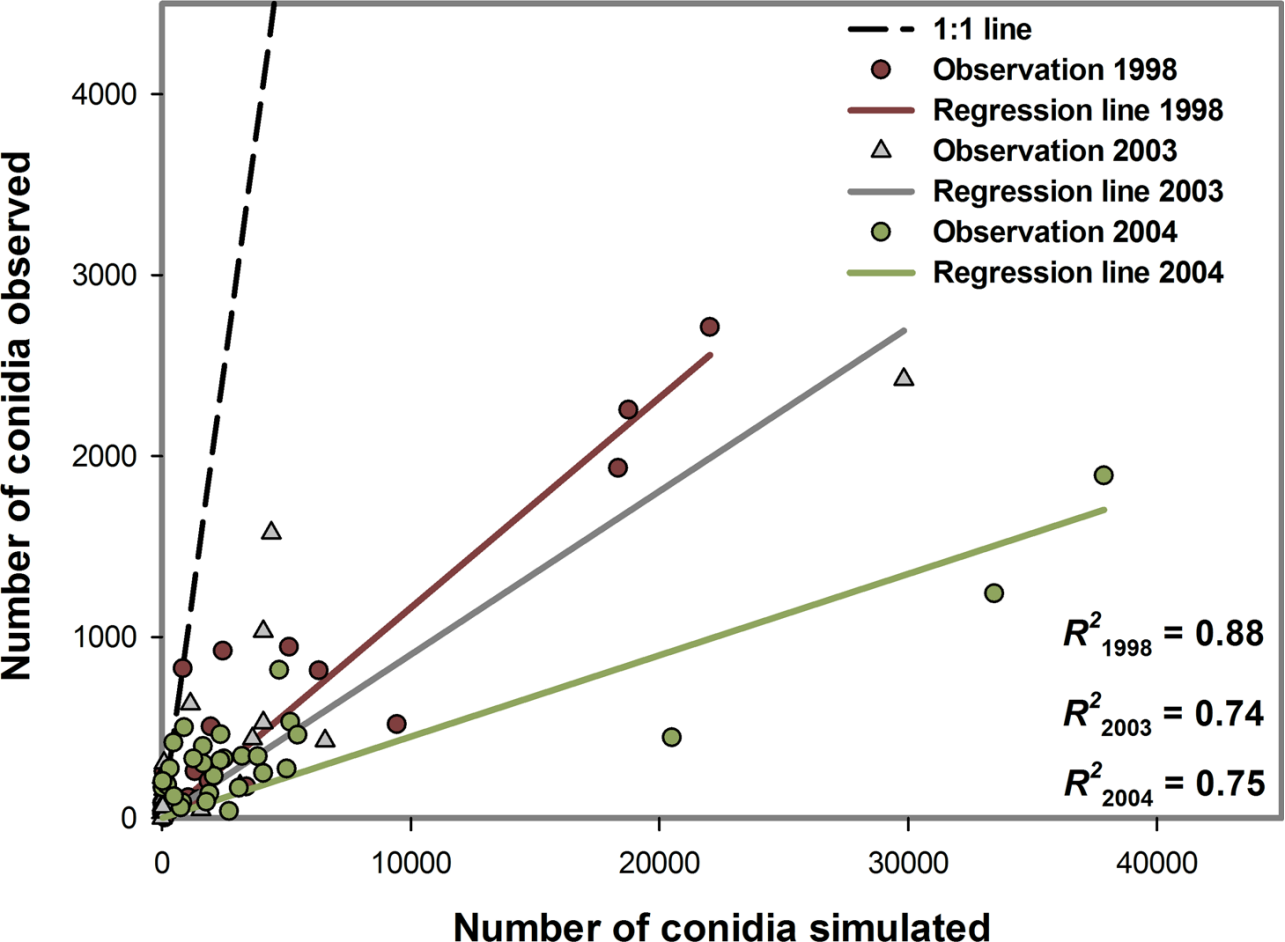
Regression analysis of daily-simulated versus daily-observed airborne conidia concentrations of *Bremia lactucae* in 1998, 2003, and 2004 for the dynamic model developed to monitor airborne inoculum of the lettuce downy mildew pathogen. The solid lines indicate the fitted values from the regressions of simulated versus observed airborne conidia. *R*^2^, coefficient of determination.

**Fig 6:**
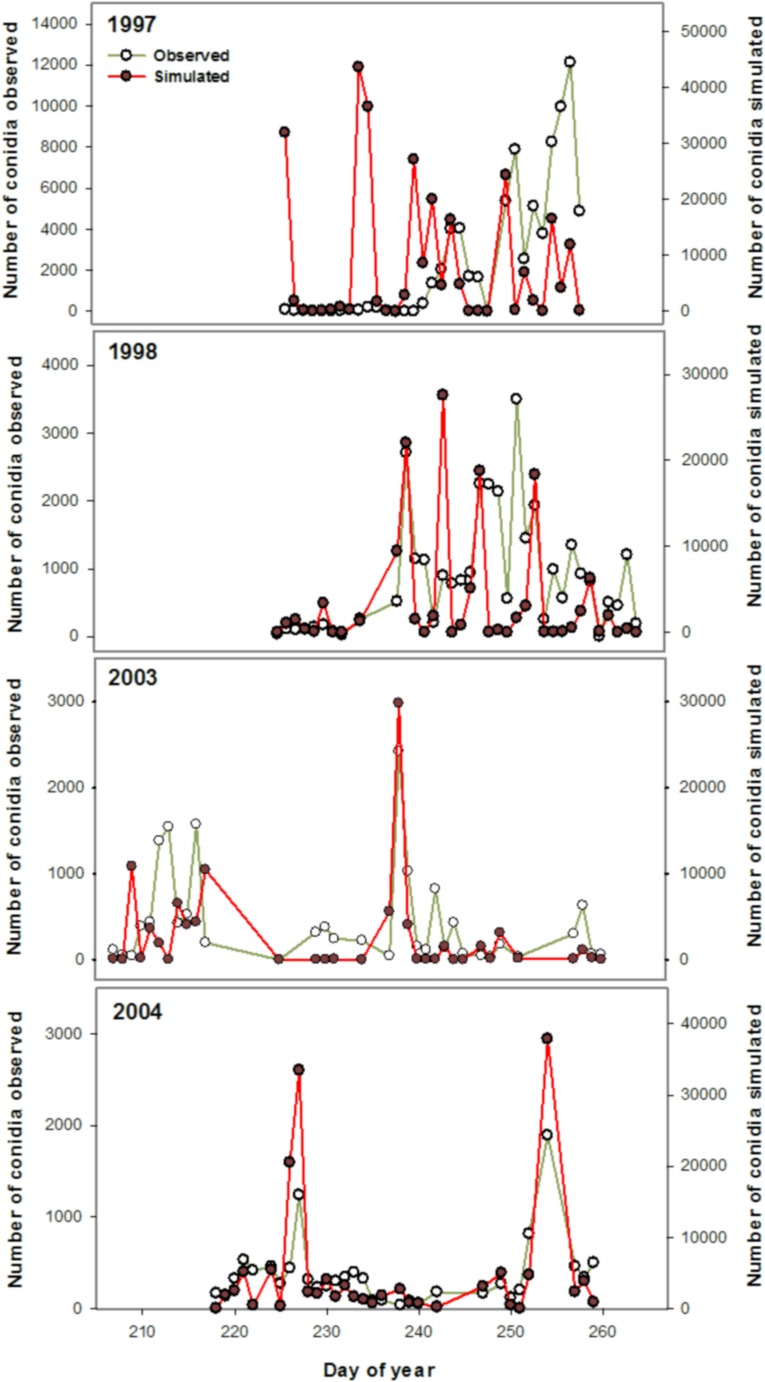
Daily-observed and daily-simulated airborne conidia of *Bremia lactucae* in 1997, 1998, 2003, and 2004 as function of day of the year (where day 1 is 1 January) for the dynamic model developed to monitor airborne inoculum of the lettuce downy mildew pathogen.

There was a linear relationship between the daily-observed number of conidia and the daily-simulated number of conidia. In 1997, 1998, 2003, and 2004, the proportions of the linear variation in the daily-observed values explained by the variation in the daily-simulated values were 0.12, 0.88, 0.74, and 0.75, respectively (Table 4, Fig 5). The trends and the conidia peaks in the daily-simulated and daily-observed conidia concentrations were similar in all years except 1997, when some simulated peaks in conidia were not observed (Fig 6).

**Table 4:** Evaluation of the regression model of daily-observed versus daily-simulated airborne conidia concentrations of *Bremia lactucae* in a lettuce field. n, number of simulations; R2, coefficient of determination; Ubias, differences between the observed and simulated airborne conidia; Uslope, deviations from the 1:1 line; Uerror, the unexplained variance.

Year	*n*	*R*^2^	U_bias_	U_slope_	U_error_
**1997**	32	0.12	-	-	-
**1998**	36	0.88	0.28	0.71	0.00
**2003**	33	0.74	0.14	0.85	0.00
**2004**	31	0.75	0.22	0.78	0.00

In 1997, as the coefficient of determination was not greater than 0.7, Theil’s decomposition of error was not performed.

**Fig 7:**
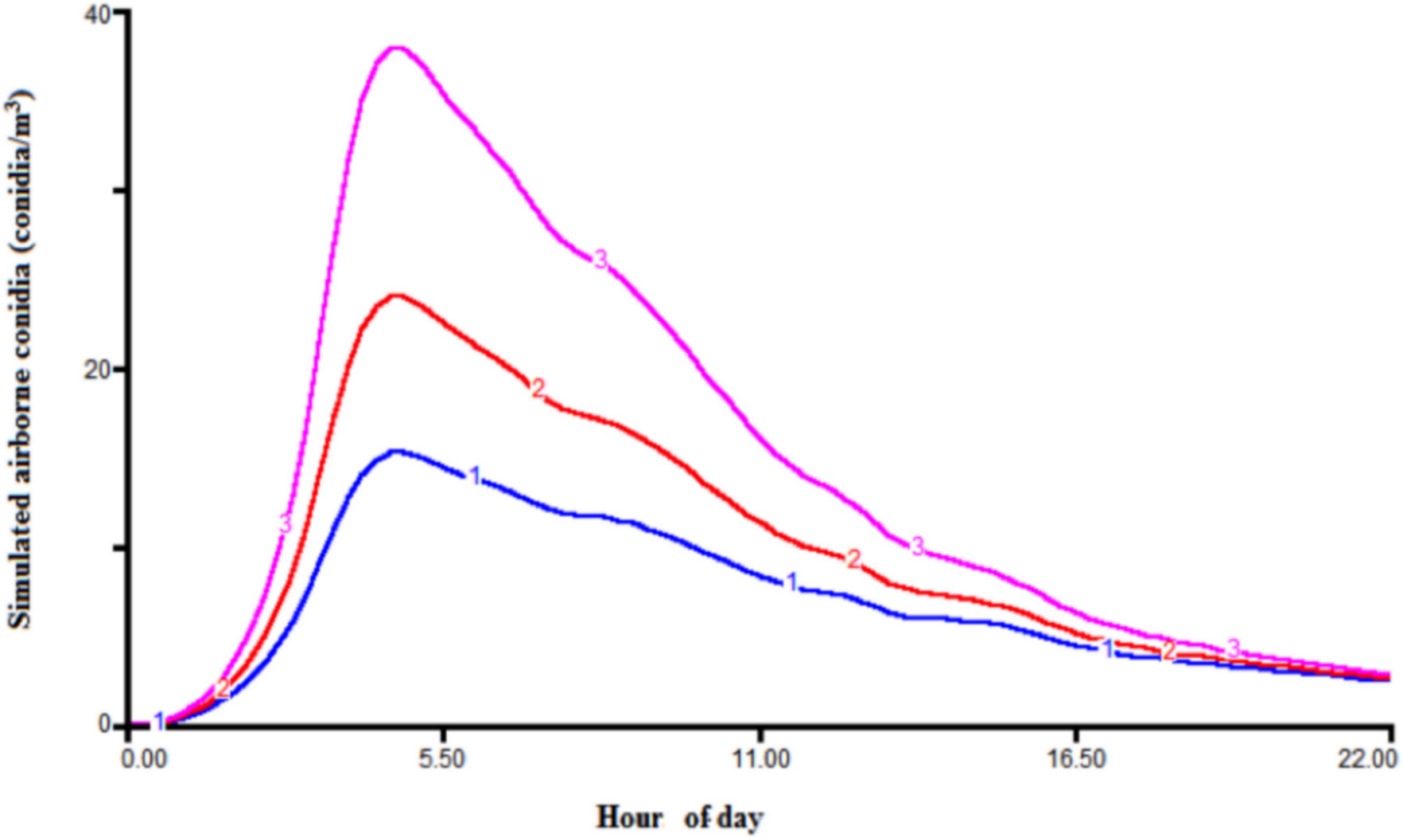
Sensitivity analysis of the model to the sporulation rate. The blue (1), red (2), and purple (3) lines represent the simulated airborne conidia for sporulation rates of 0.5, 0.6, and 0.7, respectively.

Most of the errors in the daily-simulated conidia came from the deviations from the 1:1 line. In 1998, 71% of the error was due to the deviation from the 1:1 line, whereas the proportion of error associated with the bias was 3%. In 2003, 85% of the error was due to the deviation from the 1:1 line, whereas the proportion of error associated with the bias was 14%. In 2004, 78% of the error was due to the deviation from the 1:1 line, whereas the proportion of error associated with the bias was 22% (Table 4). In 96%, 98%, 99%, and 99% of cases when no airborne conidia were measured, the daily-simulated airborne conidia concentrations were 0 in 1997, 1998, 2003, and 2004, respectively. Sensitivity analysis of the model showed that the model was highly sensitive to the rate of sporulation (r1 in the Sp equation in Table 2), which is the multiplication factor of spore production. Increasing the sporulation rate to one unit increased the simulated conidia number to 1.6 units (Fig 7). Also, the model was not significantly sensitive to other inputs variables that were tested (leaf wetness duration, wind speed, solar radiation, and RH) in this study.

## Discussion

Commercially acceptable control of lettuce downy mildew is achieved when only a few external leaves are infected. Regardless of the type of lettuce (head, leaf, or romaine), the commercialized parts must be free of disease. Because the development of lettuce downy mildew is strongly related to the environmental conditions [15], the decision support systems (DSS) that have been developed in the last 20 years rely on weather conditions to predict the best time for fungicide applications. However, epidemics are also driven by the presence and quantity of inoculum in the lettuce field [26]. Consequently, to improve the effectiveness of DSSs, it is crucial to find a way to simulate or measure airborne inoculum above the lettuce canopy [12, 31]. The results of recent studies [17, 31] suggested that inoculum could be monitored with a spore-sampling network. A high disease risk as estimated by a DSS in combination with a significant airborne spore concentration would trigger fungicide applications. However, there are advantages and limitations associated with both monitoring and simulating airborne inoculum. Spore sampling is generally representative of the real airborne inoculum concentration. However, because several samplers may be necessary to achieve an acceptable level of representativeness [17] the cost and time required to obtain the information are increased, especially when the spore count is done by microscopy. In contrast, using a simulation model to estimate airborne inoculum is less costly and easier to implement once the model has been developed. However, the reliability of simulations is influenced considerably by the quality of the weather data used. The ideal situation is probably to combine the monitoring and simulation of airborne inoculum.

In this study, a weather-based simulation model of *B*. *lactucae* ACC was developed and validated with independent data (data not used to develop the model). For this model, the asexual life cycle of *B*. *lactucae* was divided into six state variables, and the changes from one state to the next were described using mathematical equations derived from the scientific literature or developed by assembling knowledge of the interactions between pathogen and host. The model was evaluated with four years of data by comparing model simulations with field observations of hourly and daily airborne conidia concentrations.

The model followed the trend and shape of hourly and daily-observed conidia concentrations almost perfectly. In over 94% of the simulation runs, the proportion of the linear variation in the hourly-observed values explained by the variation in the hourly-simulated values was greater than 0.7 in all years except in 1997.

Over the four-year simulation period, the proportion of the linear variation in the daily-observed values explained by the variation in the daily-simulated values was greater than 0.70 in all years except in 1997. Also, most of the errors in the daily-simulated conidia came from the deviations from the 1:1 line, and the proportion of error associated with the model bias was low. Overall, the model was accurate in simulating the trend and the peaks in *B*. *lactucae* ACC, even though the model overestimated the daily number of conidia on some days. This situation is due probably to the model’s sensitivity to the sporulation rate. Indeed, sensitivity analysis showed that the model was highly sensitive to variation in the sporulation rate, which was defined as a function of temperature. Further field validations to calibrate the sporulation model developed by Tchervenivanova (1995) seem to be necessary.

These results can be useful for decision making to improve lettuce downy mildew management. Indeed, Fall et al. [15] found an exponential relationship between downy mildew intensity and the airborne conidia concentration (ACC). An ACC of 14 conidia/m^3^ was required to cause one lesion per leaf in the field [15]. Over 95% of the time, when no airborne conidia were measured, the daily-simulated ACC was 0. Therefore, when the simulated number of airborne conidia is 0, fungicides should not be applied, whereas simulated peaks in airborne conidia should lead to fungicide applications. Indeed, the disease will develop only if the pathogen is present in the area [20, 22]. In the pathosystem formed by potato and *Phytophthora infestans*, an oomycete like *B*. *lactucae*, the first signs of disease were detected between 6 and 7 d after the peak in airborne spores [17, 32]. Thus, instead of waiting for signs of downy mildew in the field before running the BREMCAST DSS [12], the model developed in this study could be used. It may be risky to wait until downy mildew is noticed in the field before running a BREMCAST DSS. Thus, DSSs can be modified to incorporate simulated or measured airborne inoculum above the lettuce canopy. However, it will be a complex process to modify these DSSs without rebuilding the entire model because, these latter were not built using a dynamic system methodology. Also, one challenge involved in implementing a spore sampling network is to know whether the number of samplers is sufficient to obtain a representative airborne conidia concentration for the targeted area. In this context, it is probably better to combine monitoring and simulation of airborne inoculum instead of increasing the number of samplers, which comes at a cost.

Nevertheless, the model missed some observed conidia on certain days. Also, some simulated peaks in conidia were not observed in 1997, and the simulations during that year were not accurate on most of the days. However. the ACC is strongly related to the number of sporulating lesions across the field, and hence it is difficult to estimate the exact ACC. The uncertainties stemming from the highly variable potential number of lesions make it difficult to quantify the airborne inoculum at a large scale. At the moment, there are no methods for quantifying the number of sporulating lesions in a given set of weather conditions. Once such methods have been developed, it will be possible to circumscribe the model at a specific scale (small or large) and improve the accuracy of quantitative simulations of airborne conidia.

### Conclusion

To the authors’ knowledge, this is the first time in the published literature that a quantitative dynamic simulation model of airborne conidia was developed. The model offers advantages for accurately simulating the trend and the temporal progression of *B*. *lactucae* airborne conidia. Indeed, from a strictly epidemiological point of view, the focus is on the rate of change rather than the direct stage of the process. Decision support system related to polycyclic disease such as lettuce downy mildew can be significantly improved by taking into account quantitative aspects of the asexual cycle of the pathogen. The existing DSSs in the literature did not take into consideration airborne conidia derived from the asexual life cycle of *B*. *lactucae*. The results obtained with this model generally compare favorably with field-observed data for airborne conidia. To the authors’ knowledge, only the PLANT-Plus model developed by Dacom for the management of *Phytophthora infestans* integrates a submodel for airborne spores. Nonetheless, this submodel does not simulate the absolute number of spores; it just confirms their presence [33]. Moreover, the model developed in the present study may be used as a research tool for investigating the impact of agricultural practices (e.g., irrigation systems) or weather conditions (e.g., temperature, wind speed, and RH) on airborne inoculum. The next step in the development of the model will be incorporating it into DSSs in order to more efficiently predict episodes of lettuce downy mildew.

## Supporting Information

S1 Dataset Observed and simulated data of airborne conidia of *Bremia lactucae*. (XLS)

S2 Dataset All STELLA equations used in the model. (DOCX)
