# Supplementary material for: A Quantitative Dynamic Simulation of Bremia lactucae Airborne Conidia Concentration above a Lettuce Canopy
Source: PLoS One. 2016 Mar 8;11(3):e0144573. doi: 10.1371/journal.pone.0144573 (PMC4783011; doi:10.1371/journal.pone.0144573)
Supplement: S2 Dataset — (DOCX) [file pone.0144573.s002.docx]

**Supporting Information:** All STELLA equations used in the model are given below.

RNSC(t) = RNSC(t - dt) + (Surviving – Deposited- UDC) * dt

INIT RNSC = 0

INFLOWS:

Surviving = RNEC*Surv_rate

OUTFLOWS:

UDC= 29/30*RNSC

Deposited = 1/30*RNSC

IPLAD(t) = IPLAD(t - dt) + (Being_infected) * dt

INIT IPLAD = 0

INFLOWS:

Being_infected = ((ACC*10*1/3)/LAI)*0.0135*0.0001

Number_of_lesion(t) = Number_of_lesion(t - dt) + (Incubation) * dt

INIT Number_of_lesion = 0

INFLOWS:

Incubation = CONVEYOR OUTFLOW

RNDC(t) = RNDC(t - dt) + (Deposited) * dt

INIT RNDC = 0

INFLOWS:

Deposited = 1/30*RNSC

RNEC(t) = RNEC(t - dt) + (Escaped - Surviving - death) * dt

INIT RNEC = 0

INFLOWS:

Escaped = RNRC*Escp_rate

OUTFLOWS:

Surviving = RNEC*Surv_rate

death = (1-Surv_rate)*RNEC

RNRC(t) = RNRC(t - dt) + (Released - Escaped - UEC) * dt

INIT RNRC = 0

INFLOWS:

Released = Sp*PSL

OUTFLOWS:

Escaped = RNRC*Escp_rate

UEC = 1-Escp_rate

PSL(t) = PSL(t - dt)

INIT PSL = IF(5< Tp<=25) AND (RH>=85) THEN 5000 ELSE 0

TRANSIT TIME = 1

CAPACITY = INF

INFLOW LIMIT = INF

RNGC(t) = RNGC(t - dt) + (Germinated - Incubation) * dt

INIT RNGC = 0

TRANSIT TIME = 240

CAPACITY = INF

INFLOW LIMIT = INF

INFLOWS:

Germinated = RNDC*Germ

OUTFLOWS:

Incubation = CONVEYOR OUTFLOW

ACC = GRAPH(TIME)

(6.00, 5.00), (6.12, 5.00), (6.23, 5.00), (6.35, 5.00), (6.46, 5.00), (6.58, 5.00), (6.70, 5.00), (6.81, 5.00), (6.93, 5.00), (7.04, 5.00), (7.16, 5.00), (7.28, 5.00), (7.39, 5.00), (7.51, 5.00), (7.62, 5.00), (7.74, 5.00), (7.86, 5.00), (7.97, 5.00), (8.09, 5.00), (8.20, 5.00), (8.32, 5.00), (8.43, 5.00), (8.55, 5.00), (8.67, 5.00), (8.78, 5.00), (8.90, 5.00), (9.01, 5.00), (9.13, 5.00), (9.25, 5.00), (9.36, 5.00), (9.48, 5.00), (9.59, 5.00), (9.71, 5.00), (9.83, 5.00), (9.94, 5.00), (10.1, 5.00), (10.2, 5.00), (10.3, 5.50), (10.4, 6.00), (10.5, 6.50), (10.6, 7.00), (10.8, 7.50), (10.9, 8.00), (11.0, 8.33), (11.1, 8.67), (11.2, 9.00), (11.3, 9.33), (11.4, 9.67), (11.6, 10.0), (11.7, 10.8), (11.8, 11.7), (11.9, 12.5), (12.0, 13.3), (12.1, 14.2), (12.3, 15.0), (12.4, 15.8), (12.5, 16.7), (12.6, 17.5), (12.7, 18.3), (12.8, 19.2), (13.0, 20.0), (13.1, 20.7), (13.2, 21.3), (13.3, 22.0), (13.4, 22.7), (13.5, 23.3), (13.7, 24.0), (13.8, 24.2), (13.9, 24.3), (14.0, 24.5), (14.1, 24.7), (14.2, 24.8), (14.3, 25.0), (14.5, 23.8), (14.6, 22.7), (14.7, 21.5), (14.8, 20.3), (14.9, 19.2), (15.0, 18.0), (15.2, 17.3), (15.3, 16.7), (15.4, 16.0), (15.5, 15.3), (15.6, 14.7), (15.7, 14.0), (15.9, 13.7), (16.0, 13.3), (16.1, 13.0), (16.2, 12.7), (16.3, 12.3), (16.4, 12.0), (16.6, 11.7), (16.7, 11.3), (16.8, 11.0), (16.9, 10.7), (17.0, 10.3), (17.1, 10.0), (17.2, 10.0), (17.4, 10.0), (17.5, 10.0), (17.6, 10.0), (17.7, 10.0), (17.8, 10.0), (17.9, 9.67), (18.1, 9.33), (18.2, 9.00), (18.3, 8.67), (18.4, 8.33), (18.5, 8.00), (18.6, 7.50), (18.8, 7.00), (18.9, 6.50), (19.0, 6.00), (19.1, 5.50), (19.2, 5.00), (19.3, 4.67), (19.4, 4.33), (19.6, 4.00), (19.7, 3.67), (19.8, 3.33), (19.9, 3.00), (20.0, 2.83), (20.1, 2.67), (20.3, 2.50), (20.4, 2.33), (20.5, 2.17), (20.6, 2.00), (20.7, 1.67), (20.8, 1.33), (21.0, 1.00), (21.1, 0.667), (21.2, 0.333), (21.3, 0.00), (21.4, 0.00), (21.5, 0.00), (21.7, 0.00), (21.8, 0.00), (21.9, 0.00), (22.0, 0.00)

Asymp1 = 0.966-0.000051*(Tp)^3 + (-15.575/(Tp)^2)

Asymp2 = 0.385 +0.054*Tp -0.0024*(Tp)^2

Escp_rate = 0.073*Ws-0.0087

Germ = Asymp2*EXP(-EXP(-r2*(LWD-2)))

LAI = 3

LWD = GRAPH(TIME)

(0.00, 3.00), (1.00, 4.00), (2.00, 5.00), (3.00, 6.00), (4.00, 7.00), (5.00, 8.00), (6.00, 9.00), (7.00, 10.0), (8.00, 11.0), (9.00, 12.0), (10.0, 0.00), (11.0, 0.00), (12.0, 0.00), (13.0, 0.00), (14.0, 1.00), (15.0, 0.00), (16.0, 0.00), (17.0, 0.00), (18.0, 0.00), (19.0, 1.00), (20.0, 2.00), (21.0, 3.00), (22.0, 4.00), (23.0, 2.00)

r1 = 0.5

r2 = -1.154 +0.327*Tp -0.011*(Tp)^2

reduction_rate = -16.43 +4.59*Tp

RH = 85

Sp = Asymp1*(1+25118.86*exp(-r1*LWD))^(-0.909)

SR = GRAPH(TIME)

(0.00, 0.00), (0.167, 0.00), (0.333, 0.00), (0.5, 0.00), (0.667, 0.00), (0.833, 0.00), (1.00, 0.00), (1.17, 0.00), (1.33, 0.00), (1.50, 0.00), (1.67, 0.00), (1.83, 0.00), (2.00, 0.00), (2.17, 0.00), (2.33, 0.00), (2.50, 0.00), (2.67, 0.00), (2.83, 0.00), (3.00, 0.00), (3.17, 0.00), (3.33, 0.00), (3.50, 0.00), (3.67, 0.00), (3.83, 0.00), (4.00, 0.00), (4.17, 0.00), (4.33, 0.00), (4.50, 0.00), (4.67, 0.00), (4.83, 0.00), (5.00, 0.00), (5.17, 0.00), (5.33, 0.00), (5.50, 0.00), (5.67, 0.00), (5.83, 0.00), (6.00, 0.00), (6.17, 0.00), (6.33, 0.00), (6.50, 0.00), (6.67, 0.00), (6.83, 0.00), (7.00, 0.00), (7.17, 0.00), (7.33, 0.00), (7.50, 0.00), (7.67, 0.00), (7.83, 0.00), (8.00, 0.00), (8.17, 0.00), (8.33, 0.00), (8.50, 0.00), (8.67, 0.00), (8.83, 0.00), (9.00, 0.00), (9.17, 0.00), (9.33, 0.00), (9.50, 0.00), (9.67, 0.00), (9.83, 0.00), (10.0, 0.00), (10.2, 0.00), (10.3, 0.00), (10.5, 0.00), (10.7, 0.00), (10.8, 0.00), (11.0, 0.00), (11.2, 0.00), (11.3, 0.00), (11.5, 0.00), (11.7, 0.00), (11.8, 0.00), (12.0, 0.00), (12.2, 0.00), (12.3, 0.00), (12.5, 0.00), (12.7, 0.00), (12.8, 0.00), (13.0, 0.00), (13.2, 0.00), (13.3, 0.00), (13.5, 0.00), (13.7, 0.00), (13.8, 0.00), (14.0, 0.00), (14.2, 0.00), (14.3, 0.00), (14.5, 0.00), (14.7, 0.00), (14.8, 0.00), (15.0, 0.00), (15.2, 0.00), (15.3, 0.00), (15.5, 0.00), (15.7, 0.00), (15.8, 0.00), (16.0, 0.00), (16.2, 0.00), (16.3, 0.00), (16.5, 0.00), (16.7, 0.00), (16.8, 0.00), (17.0, 0.00), (17.2, 0.00), (17.3, 0.00), (17.5, 0.00), (17.7, 0.00), (17.8, 0.00), (18.0, 0.00), (18.2, 0.00), (18.3, 0.00), (18.5, 0.00), (18.7, 0.00), (18.8, 0.00), (19.0, 0.00), (19.2, 0.00), (19.3, 0.00), (19.5, 0.00), (19.7, 0.00), (19.8, 0.00), (20.0, 0.00), (20.2, 0.00), (20.3, 0.00), (20.5, 0.00), (20.7, 0.00), (20.8, 0.00), (21.0, 0.00), (21.2, 0.00), (21.3, 0.00), (21.5, 0.00), (21.7, 0.00), (21.8, 0.00), (22.0, 0.00), (22.2, 0.00), (22.3, 0.00), (22.5, 0.00), (22.7, 0.00), (22.8, 0.00), (23.0, 0.00)

Surv_rate = IF SR<1 THEN 0.8 ELSE 0.6

Tp = GRAPH(TIME)

(0.00, 15.9), (1.00, 16.3), (2.00, 14.8), (3.00, 13.8), (4.00, 13.6), (5.00, 13.1), (6.00, 12.6), (7.00, 14.1), (8.00, 17.5), (9.00, 19.4), (10.0, 21.3), (11.0, 22.1), (12.0, 22.3), (13.0, 20.2), (14.0, 15.2), (15.0, 18.3), (16.0, 20.9), (17.0, 20.6), (18.0, 20.4), (19.0, 17.8), (20.0, 16.3), (21.0, 15.1), (22.0, 14.2), (23.0, 16.4)

Ws = GRAPH(TIME)

(0.00, 0.00), (1.00, 1.12), (2.00, 0.00), (3.00, 0.00), (4.00, 0.00), (5.00, 0.00), (6.00, 0.00), (7.00, 0.00), (8.00, 1.68), (9.00, 1.68), (10.0, 1.12), (11.0, 3.92), (12.0, 3.08), (13.0, 2.52), (14.0, 1.68), (15.0, 3.92), (16.0, 0.84), (17.0, 2.52), (18.0, 1.12), (19.0, 0.00), (20.0, 0.00), (21.0, 0.00), (22.0, 0.00), (23.0, 0.00)
